# Clinical and radiological comparison of percutaneous cervical nucleoplasty combined with ultrasound-guided pulsed radiofrequency of cervical nerve root for cervical radicular pain: a retrospective, matched-cohort study

**DOI:** 10.3389/fpain.2025.1618608

**Published:** 2025-07-23

**Authors:** Baodong Wang, He Song, Tianyi Wang, Peng Du, Lei Zang, Lihui Yang

**Affiliations:** Department of Orthopedics, Beijing Chaoyang Hospital, Capital Medical University, Beijing, China

**Keywords:** ultrasound guidance, cervical radicular pain, pulsed radiofrequency, percutaneous cervical nucleoplasty, outcomes

## Abstract

**Background:**

The best treatment yielding clinical benefits was still equivocal and controversial for the treatment of cervical radicular pain (CRP). This study aimed to propose a novel combination strategy of percutaneous cervical nucleoplasty (PCN) and ultrasound-guided pulsed radiofrequency (PRF) of cervical nerve root for CRP, and to compare its therapeutic effects with PRF alone.

**Methods:**

120 CRP patients who satisfied the inclusion requirements between January 2016 and March 2019 were retrospectively analyzed and split into PCN + PRF and PRF groups. The propensity score matching (PSM) technique was used to correct the imbalanced confounding variables between the groups. Then, clinical outcomes including the visual analog scale (VAS) score, Neck Disability Index (NDI) score, clinical assessment scale for cervical spondylosis (CASCS), modified MacNab criteria, radiological parameters, and complications were evaluated.

**Results:**

In all, 120 patients were used to calculate the propensity score, producing 26 matched pairs that were monitored for a minimum of a year. When compared to the preoperative data, both groups' neck pain VAS scores, arm pain VAS scores, NDI scores, and CASCS scores saw a significant improvement during the follow-up period (*p* < 0.001). However, patients in the PRF group noted higher neck pain VAS scores, arm pain VAS scores, NDI scores, and CASCS scores than those in the PRF + PCN group at the final follow-up (*p* < 0.05). The decrease in surgical level disc height was more pronounced in the PRF + PCN group at the final follow-up (*P* < 0.05). The ROM was reduced in the PRF group but increased in the PRF + PCN group at the final follow-up (*P* < 0.01). Based on the modified MacNab criteria, the PRF and PCN + PRF groups had excellent and good rates of 76.92% and 84.62%, respectively, with no statistically significant difference (*P* > 0.05).

**Conclusion:**

We present and describe a novel strategy for the combined treatment of CRP in chronic cervical radicular pain using ultrasound-guided percutaneous disc radiofrequency ablation PCN and spinal nerve root pulse radiofrequency PRF, which is both effective and safe throughout the treatment process, reducing pain and improving function.

## Introduction

Cervical radicular pain (CRP) is typically caused by degenerative disc changes, and the primary clinical manifestations are neck pain, arm pain, or both (1). According to four moderate to high-quality studies, the prevalence of CRP ranges from 1.21 to 5.8 per 1,000 (2). For the majority of cervical disc herniation patients, conservative treatment is still the preferred first step, which includes rest, physical therapy, oral medications, and epidural steroid injections (3). Pharmacological therapy can vary in effectiveness due to individual differences. Long-term use of non-steroidal anti-inflammatory drugs may cause gastrointestinal discomfort and renal impairment; the use of steroids can lead to weight gain, increased blood sugar, and osteoporosis; while opioids can effectively relieve severe pain, they carry the risk of addiction (4). Physical therapy may overlook long-term prevention, with heat therapy posing a risk of burns, and traction and massage therapies potentially exacerbating cervical spine issues if improperly performed (5). Epidural steroid injections can alleviate pain but are often temporary in effect and carry the risk of infection and nerve damage, with steroid side effects similar to those in pharmacological therapy (6). Therefore, if conservative treatment fails to provide the desired results, various percutaneous minimally invasive surgeries can be considered to relieve pain while avoiding open surgery (7–9). These minimally invasive surgeries are designed to relieve pressure or chemical irritation on sensory structures while minimizing damage to normal tissues, thus aiding patient recovery.

Percutaneous cervical nucleoplasty (PCN) is a minimally invasive procedure for treating herniated discs in the cervical, thoracic, and lumbar spines (10). The basic principle of this surgery is to reduce the volume by approximately 1 ml in a hydraulic space, such as a healthy disc, resulting in a volume reduction of about 10%–20%, significantly lowering the pressure. This decrease in pressure may alleviate the chemical and mechanical factors that contribute to symptoms such as pain, sensory loss, and motor function (11). Pulsed radiofrequency (PRF) has been used to treat various chronic pain conditions, including radicular pain, joint pain, myofascial pain, and migraines (12). During PRF treatment, the tissue temperature can reach up to 42°C, preventing the negative effects of irreversible tissue damage (9). Radiofrequency treatment near the dorsal root ganglion has been shown to cause transient sensory loss in the related dermatome, whereas pain relief may last longer, prompting the development of a minimal neurodestructive current delivery mode (13). Previous research has confirmed the effectiveness of PRF stimulation in relieving cervical radicular pain, and many clinicians are now using PRF to treat this condition (8, 14, 15).

Imaging examinations, including MRI, CT and X - ray, play a vital role in the diagnosis and efficacy evaluation of cervical disc plasma radiofrequency ablation. They can clearly display the anatomical structure of the cervical disc, the extent of the lesion, and the condition of surrounding tissues, thus providing accurate basis for pre - operative diagnosis. MRI, with its excellent soft - tissue contrast, can show in detail the protrusion of the intervertebral disc and the compression of the nerve root. CT has a unique advantage in displaying bony structures and can accurately assess the height of the intervertebral disc and the changes in bony structures. X - ray examination can dynamically observe the range of motion of the cervical spine and the overall biomechanical state (16). The results of these imaging examinations provide important background information for subsequent efficacy evaluation, helping doctors accurately assess the therapeutic effect and formulate follow - up treatment plans. In the research on the correlation between imaging parameters and clinical symptoms of cervical disc plasma radiofrequency ablation, studies have shown that there is a significant correlation between disc height, Cobb angle, SVA (sagittal vertical axis) and ROM (range of motion) and patients' clinical symptoms. For example, the recovery of disc height is closely related the to degree of pain relief (17); the changes of Cobb angle and SVA can reflect the overall biomechanical state and balance of the cervical spine (18); and the improvement of ROM is closely related to the functional recovery of patients (19). These studies provide a solid theoretical basis for the imaging evaluation part of this study, which is conducive to a more comprehensive assessment of the clinical efficacy of cervical disc plasma radiofrequency ablation.

Ultrasound-guided interventions around nerve roots have received attention for their neuromodulatory properties. In recent years, pulsed radiofrequency (PRF) and PCN under ultrasound guidance have been successfully carried out in several studies, with positive outcomes (20, 21). However, only temporary pain relief is offered by these two procedures, which are usually performed independently. To improve the safety and efficacy of CRP treatment, we employ ultrasound guidance to view cervical spine structures, avoiding major blood vessels, nerves, and other vital structures. Furthermore, we have combined PCN and PRF treatments, which successfully lessen nerve root compression and inflammation, improving clinical results. We predict that this combination treatment will lower CRP safely and efficiently.

## Methods and materials

### Patient population

We examined 120 consecutive CRP patients who received the combined approach (PCN + PRF) and PRF alone. CRP inclusion criteria were: (1) cervical root pain caused by disc herniation (pain distributed along the skin of one or more cervical roots); (2) noninvasive conservative treatment has failed for at least 6 months; (3) MRI confirmed an inclusive soft disc herniation at levels C4 to C7. The exclusion criteria are as follows: (1) previous cervical trauma or invasive cervical spine treatment; (2) Cervical spondylotic myelopathy; (3) Allergic to local anesthetics; (4) Patients who received invasive cervical spine treatment again during follow-up after intervention; (5) Patients with incomplete or missing follow-up data; (6) x-rays show that the disc height is 50% lower than normal; (7) CT or MRI examination revealed spinal stenosis, osteophyte or posterior longitudinal ligament as the main stressor, and giant disc herniation or prolapse; (8) Tuberculosis or tumor of the vertebral body. Patients who will receive the combination strategy (PCN + PRF) constitute the observation group, and those who will receive PRF constitute the PRF alone control group. Both interventions are performed in the inpatient department, and the surgeon and the patient jointly decide on the intervention following a thorough explanation and full understanding of the procedure. The study followed the principles outlined in the Declaration of Helsinki. This study was authorized by the Ethics Committee of Beijing Chaoyang Hospital. The written informed consent of all patients and their consent to review medical records were obtained before the operation.

Given the study's non-randomized nature and the various factors that could influence the outcomes, we created a propensity score matching (PSM) cohort (caliper value set at 0.02) to balance the impact of confounding variables when comparing clinical outcomes between the two groups. The propensity score for each patient was calculated as a probability using a logistic regression model that included all clinically important covariates that could affect clinical outcomes: (1) age, (2) body mass index (BMI), (3) gender, (4) age-adjusted Charlson Comorbidity Index (aCCI) (22), (5) operative segment, and (6) disease duration.

### Intervention technique

**Figure 1:**
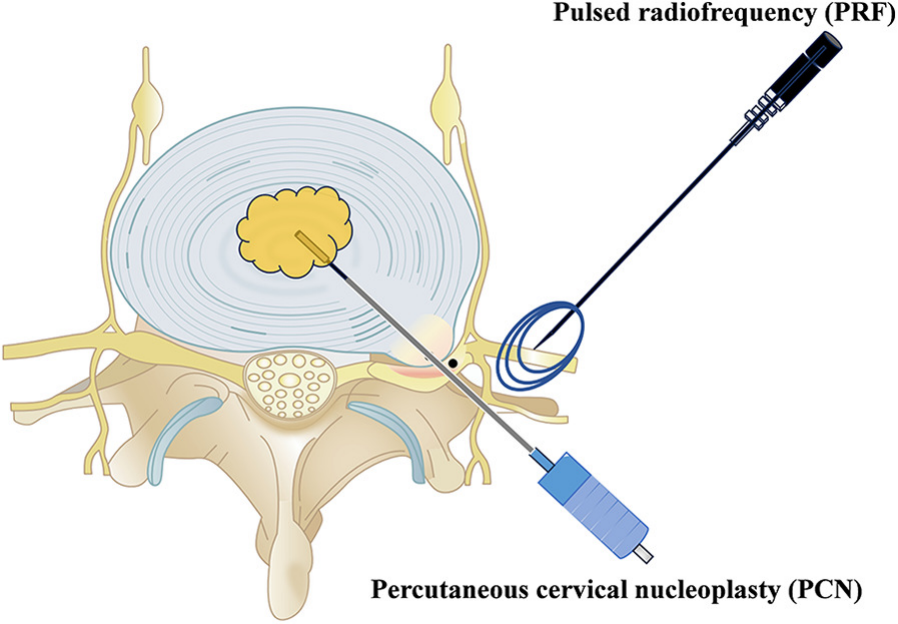
Surgical diagram of PCN and PRF.

All interventions were carried out by experienced senior orthopedic surgeons (LY, 20 years of experience). PCN used SM-D380C (Xi'an Surgical Company), while PRF used S8 EXP (Shenzhen Establishment Company). The surgical diagram is displayed in Figure 1.

**Figure 2:**
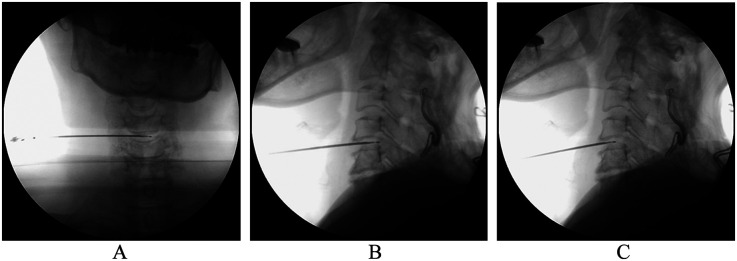
x-ray of cannula position. **(A)** Anterior–posterior and **(B)** lateral view of cannulation at initial surgical site. **(C)** The second site of laser decompression is located in the middle of intervertebral disc, confirmed by the lateral view of fluoroscopy.

PCN + PRF group: The patient was lying supine position with a soft neck cushion to stretch the neck back and keep the muscle relaxed. The C-arm x-ray machine was used to identify the vertebral space during surgery, mark the puncture point, and lay the surgical sheet after routine disinfection. The space between the carotid sheath and the visceral sheath was identified using the sterile protective cover of the ultrasound probe, which was then expanded with normal saline before performing local infiltration anesthesia with 1% lidocaine. A special puncture needle (19-gauge with a sharp tip—no stitching required) was inserted into the middle of the intervertebral space. Anteroposterior fluoroscopy was performed at the midpoint, while lateral fluoroscopy was performed on the posterior 1/3 of the intervertebral disc. The puncture needle core was removed, 2 or 3 drops of normal saline were inserted into the puncture needle sheath, and the special cold ablation cutter head for the cervical vertebra connected to the ablation host was placed under x-ray guidance to determine the cutter head's exact position. The ablation profile was set to 1 for 1 s, and the safety test was completed with no adverse reactions. The ablation gear was set to 2 for 6 s, and the intervertebral disc ablation was performed over 3 cycles: The vaporization gear was set to 1 for 1 s, and the safety test was completed without incident. Set the vaporization gear to 1 gear for 6 s and form the disc for 2 cycles: Then, set the vaporization gear to 2 gear for 6 s and form the disc for 2 cycles: Pull out the puncture needle with the cutter head for 5 m, confirm that the cutter head is in the disc using fluoroscopy, set the vaporization gear to 2 gear for 6 s, and form the fibrillar ring. In addition to two cycles, other procedures involved radiofrequency ablation of the disc. This is a controlled energy plasma-mediated process that begins with the application of radio frequency energy to a conducting medium, such as a saline solution, to generate a precisely focused plasma. The energy particles in the plasma have sufficient power to break the molecular bonds, excising or dissolving the soft tissue at relatively low temperatures (42 °C) while preserving the integrity of the surrounding healthy tissue. The plasma weld, which is approximately 120 m thick, is a conductive gas with sufficient energy to break most bonds in soft tissue molecules. Nucleus culdoplasty uses coagulation and tissue ablation to create a small hole in the nucleus pulposus (cervical spine) with the tip of a radio frequency cutter head and decompress the herniated disc (Figure 2).

**Figure 3:**
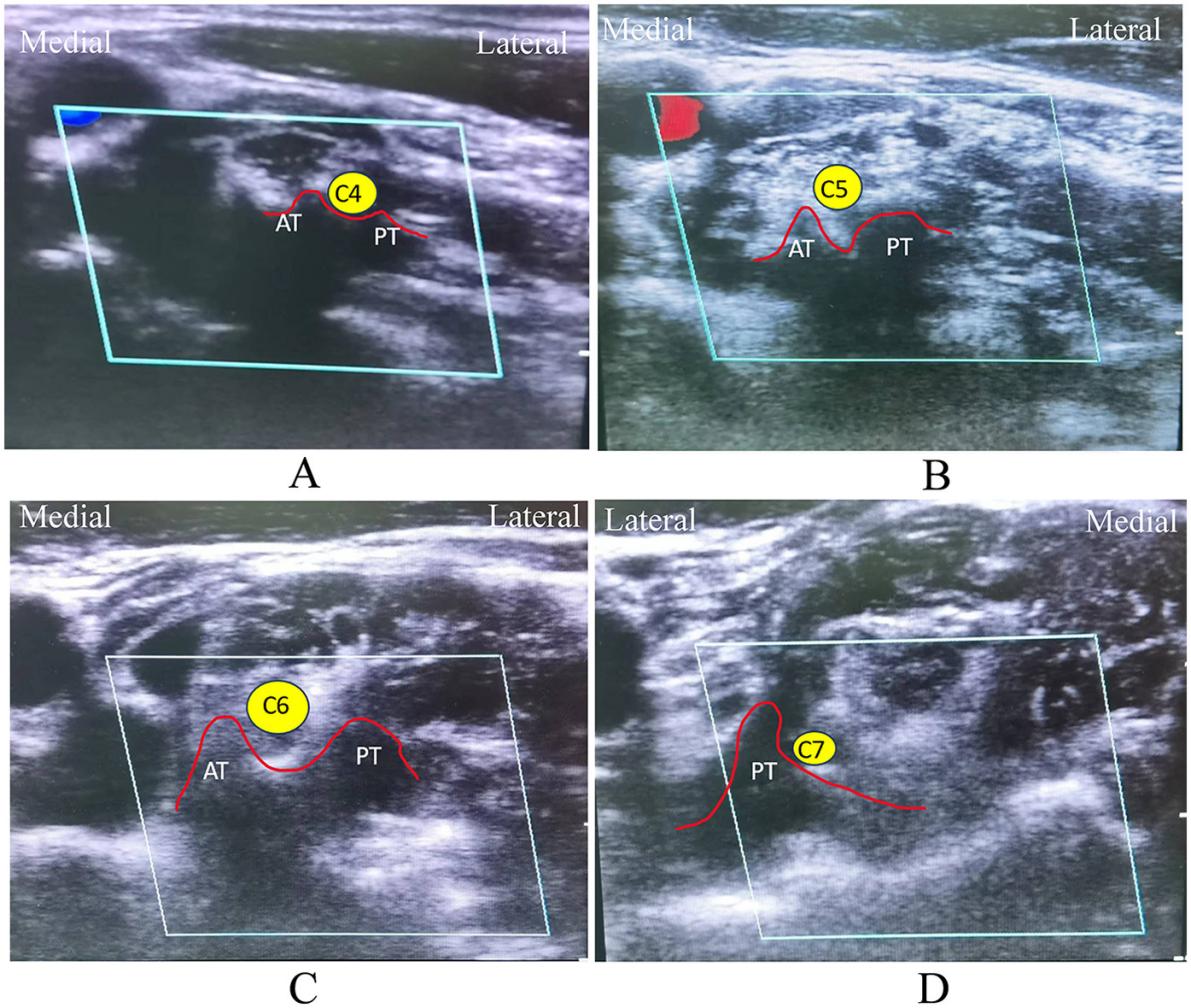
Ultrasound identification of cervical nerve roots. **(A)** Characteristics of horizontal nodules of C4 nerve root: there were two nodules in front and back under ultrasound, and the internodal sulci was shallow. **(B)** Characteristics of horizontal nodules of C5 nerve roots: there were two nodules in front and back under ultrasound, and the internodal sulci was deep. **(C)** Characteristics of horizontal nodules of C6 nerve roots: there were two nodules in front and back under ultrasound, and the anterior nodules of C6 protruded more than the posterior nodules. **(D)** Characteristics of C7 nerve root horizontal tubercle: Anterior transverse tubercle of C7 is underdeveloped, only posterior tubercle. C4: Fourth cervical nerve root, C5: fifth cervical nerve root, C6: sixth cervical nerve root, C7: seventh cervical nerve root. AT, anterior tubercle; PT, posterior tubercle.

Ultrasound localization of the anterior and posterior transverse tubercles and posterior transverse tubercles of the cervical spine during surgery, and the identification of each nerve root are shown in Figure 3. 0.5% lidocaine was injected and anesthetized layer by layer. The radiofrequency ablation needle was inserted percutaneously, and the electrode needle's location was confirmed using neurography with the ultrasonic probe's real-time guidance. The sensory and motor responses in the corresponding innerve areas were induced, and saline was injected locally, with ultrasound confirming that the drug dispersion was satisfactory. A local drug block was applied to a mixture of lidocaine, ropivacaine, and debossing, followed by pulse radiofrequency twice for each nerve at 42℃ for 120 s each.

The patient was able to resume normal activities immediately following surgery and was discharged 24 h later. Antibiotic prophylaxis was not used in every case. If required, patients receive additional conservative therapy after PCN + PRF (such as neuro edema-reducing drugs, nonsteroidal anti-inflammatory drugs [NSAIDs], or muscle relaxants). The neck collar was used for 2 weeks following surgery.

PRF group: The patient was positioned supine, and the cervical nerve roots associated with the patient's symptoms, signs, and imaging were chosen as targets. Disinfection, ultrasound-guided PRF, and postoperative treatment followed the same procedures as in the PCN + PRF group.

### Clinical outcome assessment

Data on each participant's demographic characteristics were collected. These included Age, BMI, Gender, age-adjusted Charlson Comorbidity Index (aCCI), surgical segment, and disease duration. The clinical symptoms and outcomes of all patients were assessed by reviewing their medical records and follow-up information. Follow-up assessments were conducted using regular outpatient visits for at least a year. We assessed patient-reported prognostic measures, including (1) visual analog scales (VAS-neck and VAS-arm, with scores ranging from 0 to 100, with higher scores indicating more severe pain); (2) Clinical Evaluation Scale of Cervical Spondylosis (CASCS) (the total score of CASCS is 100 points, the higher the score, the better the cervical spine function of the patients); (3) Neck Disability Index (NDI) (the total score of NDI is 50; the lower the score, the better the cervical spine function of the patient); (5) The clinical outcome was classified as excellent, good, fair, no improvement, or worse, using the modified MacNab criteria (23); (6) Safety: Complications and other safety issues during follow-up were documented. The above prognostic indexes in the combined group and PRF groups were assessed prior to surgery, 1 week, 1 month, 3 months after surgery, 6 months after surgery, and 12 months after surgery.

### Radiological assessment

In this study, both groups had one year of postoperative radiological follow-up data. We selected several imaging parameters that are closely related to cervical degeneration and pain symptoms in patients, such as sagittal x-ray images, which were used to measure disc height, Cobb's angle, and sagittal vertical axis (SVA). The range of motion (ROM) was measured using x-ray images taken at both extension and flexion. Two professional orthopedic surgeons performed all the measurements using DICOM (version 3.1) viewer software (Neusoft PACS/RIS), and the mean value was calculated. The preoperative and postoperative parameters were compared to confirm the radiological findings from PRF + PCN and PRF.

The radiological parameters were defined as follows:

(1) Disc height: the average distance between the anterior and posterior edges of the intervertebral disc at the surgical level.
(2) Sagittal Cobb's angle: The curvature of the cervical spine was indicated by Cobb's angle for C2–7 or the angle between the superior and inferior endplates that were used to assess the cervical spinal lordosis.
(3) Sagittal vertical axis: The C2–7 sagittal vertical axis (SVA), which is the distance between the plumb line from the center of the C2 to the upper-posterior edge of the C7, indicates the sagittal plane's balance.
(4) Range of motion: On the dynamic position x-ray film of the cervical spine, parallel lines were drawn along the lower endplates of C2 and the lower endplate of C7 to determine the cervical Cobb Angle in extension and flexion. The difference between the Cobb Angle in the extension and flexion positions was the cervical spine's range of motion.

### Data analysis

Statistical analyses were carried out using SPSS 24.0 (IBM Corp., Armonk, NY, USA). The paired t-test was used to compare differences in continuous variables between preoperative and follow-up clinical outcomes. For continuous data, comparisons between two groups were done using Student's *t*-test, while comparisons between multiple groups were performed using one-way ANOVA or the Kruskal–Wallis H test. The chi-square test was employed to assess categorical data. *Post hoc* comparisons were performed using the Bonferroni *post hoc* test. Statistical significance was set at a *P* < 0.05.

## Results

### Baseline characteristics before and after the PSM

120 patients met the criteria for inclusion in the propensity score calculation. The baseline characteristics of the two groups before PSM are depicted in Table 1. Covariates with SMD ≤0.2 and *P* > 0.05 were deemed balanced and comparable across both groups. However, Table 1 revealed two unbalanced covariates: age (SMD = 0.475, *P* = 0.007) and BMI (SMD = 0.357, *P* = 0.298). Following PSM, one-on-one matching yielded 26 pairs of PRF and PRF + PCN cases. Table 2 shows the baseline characteristics of both groups, where it was clear that all covariates were well-balanced and comparable.

**Table 1:** Demographic characteristics before propensity score matching.

Demographics	PRF (*n* = 47)	PRF + PCN (*n* = 73)	SMD	*P*
Age (years)	63.96 ± 13.02	57.78 ± 11.42	**0**.**475**^a^	**0**.**007**^b^
BMI (kg/m^2^)	26.05 ± 3.11	25.38 ± 3.87	**0**.**357**^a^	0.298
Gender, *n* (%)			0.029	0.86
Male	16 (34.0%)	26 (35.6%)		
Female	31 (66.0%)	47 (64.4%)		
aCCI, *n* (%)	3.33 ± 1.14	3.32 ± 1.49	0.007	0.968
Operative segment, *n* (%)			0.063	0.946
C3–4	7 (14.9%)	12 (16.4%)		
C4–5	8 (17.0%)	15 (20.5%)		
C5–6	18 (38.3%)	25 (34.2%)		
C6–7	14 (29.8%)	21 (28.8%)		
Disease duration (months)	23.64 ± 8.51	22.78 ± 7.14	0.162	0.552

PRF, pulsed radiofrequency; PCN, percutaneous cervical nucleoplasty; SMD, standardized mean difference; BMI, body mass index; aCCI, age-adjusted Charlson comorbidity index. ^a^There are varying degrees of standardized differences in the age covariate between the two groups. ^b^There is a statistical difference in the age variable between the two groups.

**Table 2:** Demographic characteristics after propensity score matching.

Demographics	PRF (*n* = 26)	PRF + PCN (*n* = 26)	SMD	*P*
Age (years)	63.15 ± 13.56	63.19 ± 13.72	0.033	0.992
BMI (kg/m^2^)	24.76 ± 3.95	24.89 ± 2.67	0.009	0.887
Gender, *n* (%)			0.021	0.569
Male	11 (42.3%)	9 (34.6%)		
Female	15 (57.7%)	17 (65.4%)		
aCCI, *n* (%)	3.38 ± 1.69	3.38 ± 1.70	0.032	0.997
Operative segment, *n* (%)			0.074	0.885
C3–4	3 (11.5%)	2 (7.7%)		
C4–5	4 (15.4%)	5 (19.2%)		
C5–6	10 (38.5%)	10 (38.5%)		
C6–7	9 (34.6%)	9 (34.6%)		
Disease duration (months)	22.15 ± 7.32	22.31 ± 7.47	0.101	0.825

PRF, pulsed radiofrequency; PCN, percutaneous cervical nucleoplasty; SMD, standardized mean difference; BMI, body mass index; aCCI, age-adjusted Charlson comorbidity index.

### Clinical outcomes

**Figure 4:**
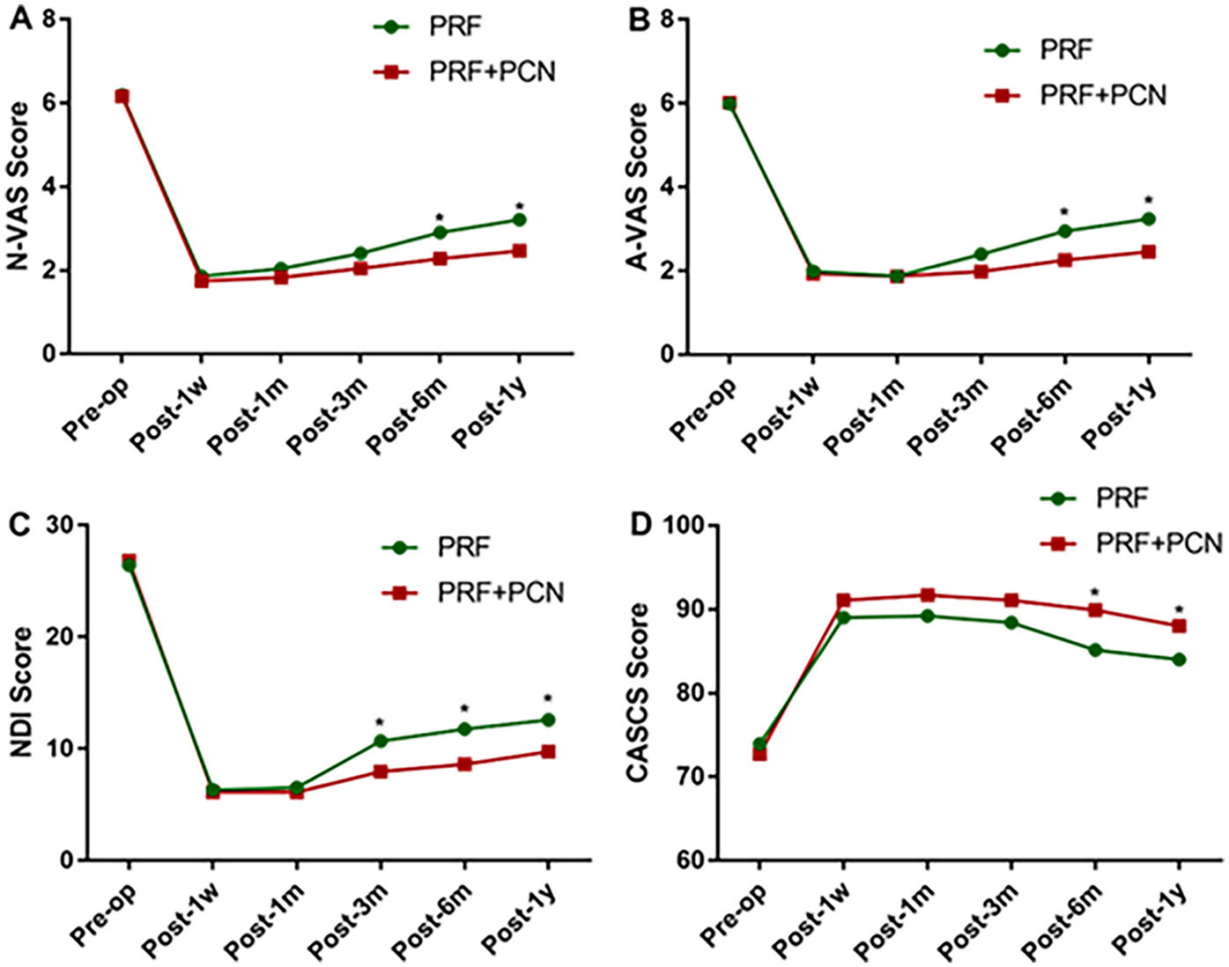
Results of the clinical efficacy of the functional scores. **(A)** Change in A-VAS scores over time. **(B)** Changes in the N-VAS scores over time. **(C)** Changes in NDI scores over time. **(D)** Changes in CASCS scores over time. A-VAS, arm visual analog scale; N-VAS, neck visual analog scale; NDI, neck disability index; CASCS, neck disability index. * Indicates a significant difference between the two groups.

As shown in Figure 4, there were no significant differences in baseline VAS, NDI, or CASCS scores between the groups. After treatment, pain and functional outcome assessments showed significant improvements in the VAS, NDI, and CASCS scores for the PCN group from 1 month to 1 year compared to baseline (*P* < 0.001 for VAS, NDI, and CASCS). There was no significant difference between the two groups in VAS, NDI, and CASCS scores one week and one month after surgery. Three months after surgery, the VAS and CASCS scores showed no significant difference between the two groups. However, the NDI scores showed a significant difference (*P* < 0.05) between the two groups. Between the 6-month and 1-year follow-ups, there was a significant difference (*P* < 0.05). Moreover, during the 1-year follow-up period used to examine therapeutic effects, PRF + PCN treatment significantly outperformed CT in terms of pain relief and functional improvements (*P* < 0.05 for VAS, NDI, and CASCS).

**Figure 5:**
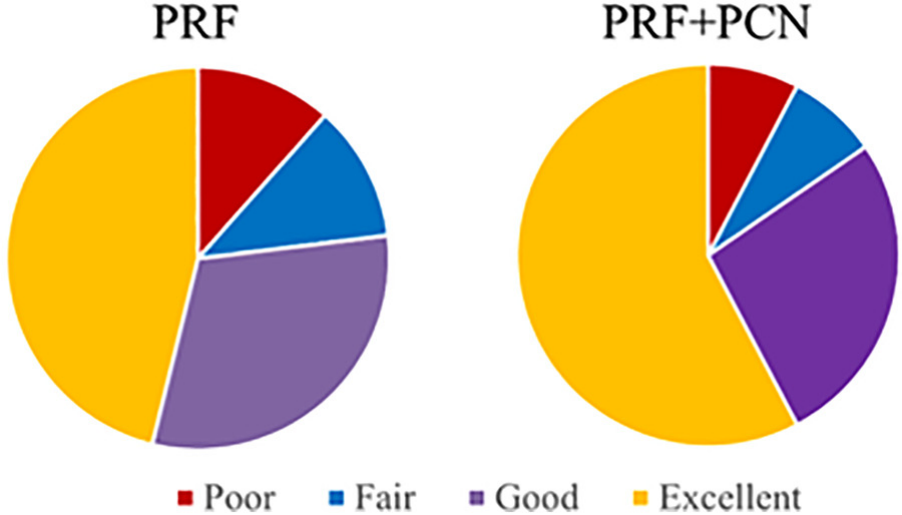
Clinical outcomes of the PRF and PRF + PCN groups at the last follow-up.

In both groups, no adverse events were directly related to the procedure. (e.g., headache, dysphagia, hemorrhage, infection, or cerebrospinal fluid leak). At the final follow-up, three patients in the PRF group indicated recurrent and intolerable neck pain with fatigue, while two patients in the PRF + PCN group reported moderate pain. According to the modified MacNab criteria, clinical satisfaction was attained in 20 (76.92%) and 22 (84.62%) patients in the PRF and PRF + PCN groups, respectively, with little difference between the two groups (Figure 5 *P* = 0.482).

### Radiological outcomes

Table 3 shows the preoperative and postoperative radiological changes. Disk height of the surgical level, C2–7 Cobb's angle, C2–7 SVA, and ROM were all measured before and after surgery in both groups. These parameters showed no significant differences between the two groups prior to surgery. In addition, after a year of follow-up, the PPF + PCN group had significantly lower surgical level disc height compared to the preoperative period, with a statistically significant difference from the PRF group (*P* < 0.05). The other parameters had no significant difference between pre-and postoperative values in patients from the two groups. However, the PRF + PCN group had significantly different ROM than the PRF group. (*P* < 0.01).

**Table 3:** Changes in radiographic parameters in the PRF and PRF + PCN groups.

Radiological parameters	PRF group (*n* = 26)		PRF + PCN group (*n* = 26)	
	Pre-operative	Final follow-up	Pre-operative	Final follow-up
Disc height (mm)	6.11 ± 1.07	6.07 ± 1.14	6.03 ± 0.76	5.65 ± 0.68^*†^
Cobb's angle (°)	10.04 ± 4.35	10.13 ± 4.77	10.32 ± 4.27	11.67 ± 4.66
SVA (mm)	9.54 ± 4.36	10.46 ± 4.71	9.67 ± 5.32	11.54 ± 7.27
ROM (degrees) (°)	6.52 ± 1.96	5.13 ± 1.82	6.60 ± 1.67	6.79 ± 1.70^†^

* Significantly different from the preoperative parameters (*P* < 0.05).

^†^ Significantly different from the PRF group Final follow-up (*P* < 0.05).

PRF, pulsed radiofrequency; PCN, percutaneous cervical nucleoplasty; SVA, sagittal vertical axis; ROM, range of motion.

## Discussion

Cervical radicular pain, also known as radicular cervical spondylosis, is the result of cervical nerve root compression. Common symptoms include neck and shoulder pain, as well as radiating pain, numbness, and weakness in the upper extremities (1). This disease is more common in middle-aged people, but in recent years, it has been gradually getting younger, with an incidence ranging from 3.8% and 17.6%, accounting for 60%–70% of cervical spondylosis patients (24). Although most patients can benefit from conservative treatment, such as medication, traction, and physiotherapy, some patients whose conservative treatment is not satisfactory, and require additional treatment options (10, 25, 26). For these patients, two minimally invasive treatment techniques, PCN (27) and spinal nerve root pulsed radiofrequency (PRF) (28) provide new treatment options. PCN reshapes the nucleus pulposus and reduces disc pressure by precisely controlling thermal energy (typically 40℃–70°C) (29), whereas PRF regulates the nervous system and relieves pain through intermittent electrical stimulation (30). These two technologies, which are minimally invasive, safe, and effective, open up new treatment options for patients.

PRF is an advanced neuromodulation technology used to treat chronic pain. The treatment works by delivering pulsed electrical currents to specific nerve roots or dorsal root ganglia, resulting in a local high-voltage field that indirectly activates superficial neurons in the spinal dorsal horn. This process alters nerve sheath cell function, inhibits electrophysiological conduction of nerve fibers, disrupts pain signal transmission, and activates the spinal cord's pain inhibition system (31). Although PRF can significantly reduce pain in the short term, its long-term efficacy may be less than that of direct nerve destruction methods, necessitating repeated treatments to manage pain (12, 31). Furthermore, the efficacy of PRF varies by individual and may be limited against certain types of pain (32). PCN, with its minimal invasiveness, wide application, and clear therapeutic outcomes, offers an alternative to overcome the limitations of PRF (20).

PCN is a minimally invasive surgical procedure that treats cervical disc herniation. It uses low-temperature plasma technology to create a highly ionized plasma region around the electrode via a conductive medium, which severs the molecular chains within the disc tissue, resulting in tissue vaporization and contraction (33). The procedure is performed at relatively low temperatures (typically 40℃–70℃) to prevent thermal injury to surrounding tissues (29). By ablating the protruding portion of the disc, the volume of the disc is reduced, relieving pressure on the nerve roots. PCN has several advantages over open cervical surgery, including minimal invasiveness, quick recovery, fewer complications, and the preservation of spinal structural integrity (16, 34). Furthermore, de Rooi j et al. (35) demonstrated that among patients with single-level cervical disc herniation, there was no statistically significant difference in symptom relief and patient satisfaction between the ACDF and PCN groups one year after surgery. Furthermore, unlike PRF, PCN uses radiofrequency ablation to directly target the protruding part of the cervical disc, effectively reducing disc compression on the nerve roots (21). This direct physical ablation can quickly relieve the symptoms of nerve root compression caused by disc herniation, whereas PRF primarily relieves pain by modulating nerve signal conduction, with less direct impact on the disc structure (8). Next, PCN can repair the annulus fibrosus of the disc using radiofrequency ablation, increasing the disc's structural stability. Conversely, PRF primarily relieves pain by modulating nerve signal transmission, with only minor reparative effects on disc structure (21).

Combining the insights from the aforementioned literature, PCN can effectively address some of the current limitations of PRF. In our clinical practice, we choose between PCN + PRF combination therapy and standalone PRF treatment for chronic cervical radicular pain based on the unique circumstances of each patient. However, no direct comparison of efficacy between these two approaches has yet been published in the clinical literature. Therefore, we chose 26 pairs of patients with CCRP to undergo the two treatment methods described above. The study findings revealed that the NDI of patients in the PCN + PRF group was considerably lower than that of the PRF group after 3 months, the N-VAS and A-VAS scores of patients in the PCN + PRF group were substantially less than those of the PRF group after 6 months, and the CASCS scores of patients in the PCN + PRF group were significantly greater than those of the PRF group after 6 months. The study findings show that, when compared to the PRF group alone, the combined treatment did not show significant differences in the short term, but its benefits gradually emerged in the later stages of treatment (after 3 months), and the variations in therapeutic effects increased over time. Furthermore, based on the modified MacNab criteria, a greater proportion of patients in the PRF + PCN group reported clinical satisfaction than those in the PRF group. The reason could be that PCN acts directly on the intervertebral disc to eliminate the source of compression, whereas PRF reduces pain signals through neural modulation. The combined treatment addresses structural and functional issues, resulting in more comprehensive and effective relief of cervical radicular pain (28). Furthermore, PCN can quickly relieve symptoms in the short term, whereas PRF has long-term analgesic effects. This complementary action enables the combined treatment to provide effective therapeutic results at various stages (15).

In terms of imaging assessment, after a year of follow-up, the intervertebral disc height at the surgical level in the PCN + PRF group was significantly lower than preoperatively. A case–control study (16) found a significant decrease in average intervertebral disc height from 6.04 ± 0.85 mm to 5.76 ± 1.02 mm 3 months after PCN treatment, which is consistent with our findings. The study also found that the amount of intervertebral disc height reduction was unrelated to the significant improvement in visual analog scale (VAS) scores. The reason for the decrease in intervertebral disc height can be summarized as PCN ablation of intervertebral disc tissue, which results in a decrease in disc volume and, as a result, a decrease in disc height. Furthermore, our study discovered that a decrease in intervertebral disc height did not result in the development of new clinical symptoms. Furthermore, when compared to the PRF group, the ROM of the PCN + PRF group increased. Possible explanations include PCN ablation of intervertebral disc tissue using low-temperature plasma technology, which results in a reduction in disc volume (36). This volume reduction alters the biomechanical properties of the cervical spine, reducing the disc's constraining force on adjacent vertebrae and increasing disc mobility. Second, after PCN treatment, the reduction in intervertebral disc height narrows the intervertebral space, which affects cervical spine stability and increases disc mobility (16). Next, PRF regulates nerve signal conduction, which prevents abnormal nerve discharge. By relieving nerve root pain and inflammation, patients may increase their cervical spine's range of motion in a pain-free state, thereby increasing disc mobility (37).

Our study has some limitations. First, it used a retrospective single-center design with a small sample size, making it impossible to rule out selection bias. Future research should include larger-scale prospective, multicenter studies to further validate our findings. Second, because we did not establish a control group of patients who received only PCN treatment, we cannot determine whether the benefit of long-term postoperative pain relief is due to the PCN technology itself or the synergistic effect of PCN combined with PRF treatment. This requires additional well-designed studies to prove. Finally, our sample may not have included all types of cervical radicular pain patients, such as those with varying causes, disease stages, ages, and genders. This may result in differences in the applicability of research findings across patient groups, making it difficult to comprehensively evaluate the efficacy of PCN + PRF combined treatment vs. PRF treatment alone in different types of patients. In future studies, we will refine the types of cervical spondylotic radiculopathy to reach more precise conclusions.

## Conclusion

We introduce a novel approach for treating CRP by combining PCN with PRF. This integrated strategy is both effective and safe, consistently reducing pain levels and improving functional outcomes throughout the treatment process.

## Data Availability

The raw data supporting the conclusions of this article will be made available by the authors, without undue reservation.
